# Verification of effectiveness of cognitive function test using Cognitive Impairment Screening Test (CIST)

**DOI:** 10.1002/alz70860_102562

**Published:** 2025-12-23

**Authors:** Hojin Choi, Yangki Minn, Im‐Seok Koh, Hyuk Sung Kwon

**Affiliations:** ^1^ Hanyang University Guri Hospital, Guri, gyeonggi‐do, Korea, Republic of (South); ^2^ Kangnam Sacred Heart Hospital, Seoul, Seoul, Korea, Republic of (South); ^3^ National medical center, Seoul, Korea, Republic of (South); ^4^ Hanyang University College of Medicine, Hanyang University Guri Hospital, Guri, Korea, Republic of (South)

## Abstract

**Background:**

South Korea is entering an aging society faster than any other country in the world. As the proportion of degenerative diseases among the elderly increases, the socioeconomic costs for dementia patients and their caregivers are also rising rapidly. In response, the early dementia screening project was launched in 2007; however, cognitive function screening still faces many limitations. To address these challenges, in 2020, a study was initiated to develop a Korean screening tool that is easy to use in the national dementia screening program and has excellent discriminatory power for cognitive impairment, resulting in the development of the Cognitive Impairment Screening Tool (CIST). This study aims to verify the appropriateness and effectiveness of the screening tool by implementing the national dementia screening program over three years (2021–2023) using the standard administration guidelines for the CIST.

**Method:**

From January 2017 to December 2023, individuals registered in the Korea Central Dementia Center database as part of the early dementia screening program were selected as study subjects. Cognitive impairment was determined through the Korean version of the Consortium to Establish a Registry for Alzheimer's Disease (CERAD) test and subsequent specialist consultation. The study aimed to assess the degree of agreement between the presence or absence of cognitive impairment identified by the Cognitive Impairment Screening Tool (CIST) and existing tests, and the clinical diagnosis (through diagnostic and differential diagnostic tests) made by a specialist. In addition, sensitivity, specificity, positive predictive value, and negative predictive value were compared and evaluated.

**Result:**

The total number of patients included in the analysis was 4,161,475, and the total number of tests analyzed was 7,851,821. Compared to the existing screening test, the CIST had a lower mean score and a wider score distribution. In predicting cognitive impairment, the CIST (sensitivity: 83.0%, specificity: 44.8%, positive predictive value: 92.9%, negative predictive value: 23.2%) was deemed overall more effective than the existing screening test (sensitivity: 81.0%, specificity: 44.5%, positive predictive value: 92.8%, negative predictive value: 20.8%).

**Conclusion:**

Compared to existing screening tests, the CIST appears to have greater effectiveness in identifying individuals with cognitive impairment during diagnostic testing.

